# Spatio-temporal analysis to identify determinants of *Oncomelania hupensis* infection with *Schistosoma japonicum* in Jiangsu province, China

**DOI:** 10.1186/1756-3305-6-138

**Published:** 2013-05-06

**Authors:** Kun Yang, Wei Li, Le-Ping Sun, Yi-Xin Huang, Jian-Feng Zhang, Feng Wu, De-Rong, Hang, Peter Steinmann, You-Sheng Liang

**Affiliations:** 1 Jiangsu Institute of Parasitic Diseases, Wuxi, China; 2 Key Laboratory on Technology for Parasitic Disease Prevention and Control, Ministry of Health, Wuxi, China; 3 Department of Epidemiology and Public Health, Swiss Tropical and Public Health Institute, Basel, Switzerland; 4 University of Basel, Basel, Switzerland

**Keywords:** Schistosomiasis, Infected snails, Determinants, Spatio-temporal analysis, China

## Abstract

**Background:**

With the successful implementation of integrated measures for schistosomiasis japonica control, Jiangsu province has reached low-endemicity status. However, infected *Oncomelania hupensis* snails could still be found in certain locations along the Yangtze river until 2009, and there is concern that they might spread again, resulting in the possible re-emergence of infections among people and domestic animals alike. In order to establish a robust surveillance system that is able to detect the spread of infected snails at an early stage, sensitive and reliable methods to identify risk factors for the establishment of infected snails need to be developed.

**Methods:**

A total of 107 villages reporting the persistent presence of infected snails were selected. Relevant data on the distribution of infected snails, and human and livestock infection status information for the years 2003 to 2008 were collected. Spatio-temporal pattern analysis including spatial autocorrelation, directional distribution and spatial error models were carried out to explore spatial correlations between infected snails and selected explanatory factors.

**Results:**

The area where infected snails were found, as well as their density, decreased significantly between 2003 and 2008. Changes in human and livestock prevalences were less pronounced. Three statistically significant spatial autocorrelations for infected snails were identified. (i) The Moran’s I of infected snails increased from 2004 to 2007, with the snail density increasing and the area with infected snails decreasing. (ii) The standard deviations of ellipses around infected snails were decreasing and the central points of the ellipses moved from West to East. (iii) The spatial error models indicated no significant correlation between the density of infected snails and selected risk factors.

**Conclusions:**

We conclude that the contribution of local infection sources including humans and livestock to the distribution of infected snails might be relatively small and that snail control may limit infected snails to increasingly small areas ecologically most suitable for transmission. We provide a method to identify these areas and risk factors for persistent infected snail presence through spatio-temporal analysis, and a suggested framework, which could assist in designing evidence based control strategies for schistosomiasis japonica elimination.

## Background

Schistosomiasis japonica is a zoonotic disease caused by an infection with *Schistosoma japonicum*. *Oncomelania hupensis* serves as the intermediate host snail of *S. japonicum*[1,2]. Previous studies have shown that *O. hupensis* in China is mainly distributed along the Yangtze river valley and in southern China. The distribution of *S. japonicum* is much more restricted [3]. Snails are infected when they are penetrated by miracidia, the larval stage of *S. japonicum* hatching from eggs when they reach water after being deposited with feces from the mammalian definitive hosts [4,5]. In the People’s Republic of China, approximately 65 million individuals are currently at risk of infection with *S. japonicum*[6-8]*.*

**Figure 1:**
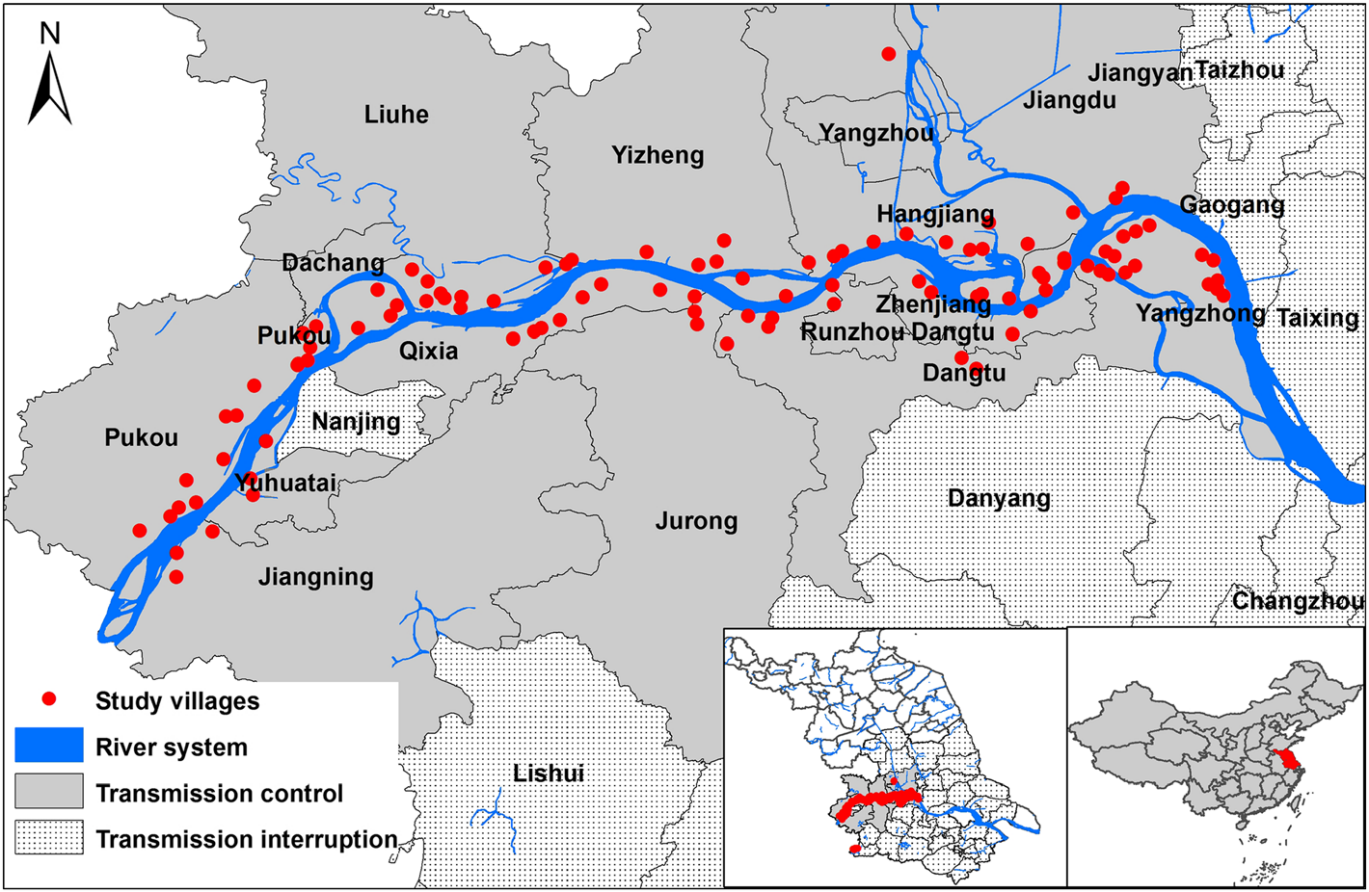
**Location of the study area in Jiangsu province, China, and distribution of study villages.** The schistosomiasis japonica control situation is described as transmission interruption (no snail is found) or transmission control (no acute case of schistosomiasis is found).

Jiangsu province is located on the lower reaches of the Yangtze river on the East coast of China (Figure 1). Flooding caused by the Yangtze river continues to be a prime risk factor for schistosomiasis in China [9,10]. It impacts on local disease endemicity, the number of acute cases and the geographic areas where infected snails are found as it increases their habitat [11-14]. In Jiangsu province, more than 90% of all current snail habitats are found along the shore of the Yangtze river. By the end of 2004, a total of 213,000 ha of habitat containing infected snails and 39 acute human schistosomiasis japonica cases were reported [10,15]. In 2005, the provincial government strengthened the implementation of integrated measures that aimed to reduce the transmission of *S. japonicum*[16]. In the wake of the programme, the average prevalence among humans decreased from 0.70% in 2005 to 0.11% in 2008, and among livestock from 0.019% to 0. Furthermore, no infected snails were found in the province from 2009 onwards. The surveillance system put in place to monitor the situation focuses mainly on humans and intermediate hosts. However, the determinants of infected snail occurrence requires further study in order to identify relevant risk factors and efficiently prevent the re-emergence of infected snail populations. Several studies have demonstrated that snails were always present in clusters [17,18]. The application of spatial technology, including geographical information systems (GIS), remote sensing (RS) and spatial statistics for schistosomiasis research since the 1990s has resulted in important advances in our understanding of the key factors determining schistosomiasis transmission [2,19-21]. Recently, the application of directional distribution analysis became more common in many study fields, for example the mapping of crime hotspots where it might identify a relationship between the distribution and trends of criminal activity and particular physical attributes (e.g. a particular street section) [22].

In the study presented here, we analyze the relationship between infected snail populations and local infection sources, including people and livestock in Jiangsu province based on spatio-temporal – including directional distribution – analysis, to identify the main source of infection, and explore the environmental determinants of *O. hupensis* infection.

## Methods

### Study area

The study focused on the marshland along the Yangtze river in Jiangsu province where 107 villages (Figure 1) were selected based on the following criterion: at least one infected snail had been found around the village, including in marshlands along the Yangtze river and on beaches of the rivers connected to the Yangtze river, between 2003 and 2008. The geographical co-ordinates (latitude/longitude) of each village were recorded by a GPS unit (Garmin Map76).

Between 2003 and 2008, annual surveillance covering both the human population and livestock had been carried out within the study area. In each of the study villages, all heads of livestock were examined using the standard miracidia hatching method [23]. Among humans, 90% of the individuals aged between 6 and 60 years were screened for schistosomiasis japonica infection using a serological test (Dipstick dye immunoassay, DDIA) [24]. Stool samples were then collected from individuals with positive test results to conduct the Kato–Katz thick smear test [23]. A single dose of praziquantel at a dosage of 40 mg/kg body weight was offered to all seropositive individuals, and a two-day course of praziquantel at 60 mg/kg body weight was administered to those with a positive Kato-Katz thick smear test.

Snail collection was conducted by systematically sampling with a square frame of 0.11 m^2^ that was set every 30 meters in known snail habitats. All snails inside the frame were collected. Additionally, environmental (purposeful) sampling was employed in spring and autumn of each year to detect snails in potential snail habitats in grasslands and marshlands, e.g. where snails had been detected over the last three years, in previously flooded areas etc. Systematic sampling was then carried out if any snails were found in these potential habitats. All collected snails were counted, crushed and examined by microscopy to detect sporocysts and cercariae. Various outcome indices were considered, including the *S. japonicum* prevalence among humans*,* the rate of *S. japonicum* infection in snails*,* the density of living snails, and the density of infected snails.

### Statistical analysis

Descriptive analysis was performed using the statistical software package SPSS (Version 11, SPSS Inc. Chicago, IL, USA). The analysis focused on the yearly data for *S. japonicum* infection among snails and the infection status of the human and livestock populations.

The spatio-temporal pattern analysis was carried out using the spatial analyst module of ArcGIS 10.0 (ESRI, Redlands, CA, USA) and GeoDA 1.0.1 (The GeoDa Center for Geospatial Analysis and Computation). The global Moran’s I was used to measure spatial autocorrelation in infected snail, human and livestock populations in each year. The spatial autocorrelation was used to evaluate whether the pattern was clustered, dispersed, or random. A Z score was considered for evaluating the significance of the Moran’s I value. The differences of spatial autocorrelation in each year were then used to explore spatio-temporal patterns.

Directional distribution, namely the Standard Deviational Ellipse (SDE), was used to measure the directional trend each year, and to provide information about dispersion of the infected snails, humans and livestock in terms of compactness and orientation. Employing the method was inspired by its wide application in diverse studies [25]. For example, plotting ellipses for a disease outbreak over time may be used to model its spread [26]. The distributional trend analysis can create an elliptical polygon; the attributed values for these output ellipse polygons include two standard distances (long and short axes) and the orientation of the ellipse. We used one standard deviation to represent the distribution that covers approximately 68 percent of all input variables for both the infected snails, humans and livestock [27,28]. A series of additional measurements and data including axial ratios, and co-ordinates of each ellipse in each year were collected to compare the spatial patterns of infected snails and local infection sources.

We used a spatial autoregressive error model (a spatial regression model including a spatial autoregressive error term) implemented in GeoDA 1.0.1 to measure the relationships between the density of infected snails and the serological or stool prevalence of people and livestock. Initially, we fit the data in an ordinary least squares (OLS) regression model. As expected, the results suggested considerable non-normality and heteroscedasticity, which did not satisfy the basic hypothesis of standard linear regression, as well as high spatial correlation. Based on this result we concluded that a spatial error model was more appropriate for this dataset.

Formally, this model is *y = Xβ + ϵ*, with *ϵ = λW + μ*, where *y* is a vector of observations of the dependent variable, *W* is the spatial weights matrix, *X* is a matrix of observations of the explanatory variables, *ϵ* is a vector of spatially auto correlated error terms, *μ* is a vector of i.i.d. errors, and *λ* and *β* are parameters.

### Ethics statement

The study protocol was approved by the Ethics Review Committee of the Jiangsu Institute of Parasitic Diseases, Wuxi, China. Written informed consent had also been obtained from each participant or a literate relative during the screening for infections. No specific permits were required for the field studies focusing on snails as they did not involve endangered or protected species.

## Results

Table 1 summarizes the data related to infected snails. The area and density of infected snails were 1272.686 ha and 0.015 per m^2^ in 2003, which then decreased to 97.186 ha and 0.003 per m^2^ in 2008, a decrease of 92.4% and 76.3%, respectively. The human sero-prevalence was 1.649% and 1.304% in 2003 and 2008, respectively, a non-significant decrease (*χ*^2^=7.538, df=1, P=0.06). The stool prevalence was 4.290% and 4.977% in 2003 and 2008, respectively, again not significantly different (*χ*^2^=1.138, df=1, P=0.710). Among 4714 heads of livestock examined, 21 were positive, translating into a prevalence of 0.445% over the study period. No positive livestock was found in 2008. However, the difference was again not significant (*χ*^2^=0.556, df=1, P=0.273).

**Table 1:** The characteristics of schistosomiasis japonica in the study villages of Jiangsu province, China, from 2003 to 2008

**Year**	**Area of infected snail habitat (ha)**	**Density of infected snails (/0.1m2)**	**Human sero-examination**		**Human stool examination**		**Livestock examination**	
			**No. examined**	**Prevalence (%)**	**No. examined**	**Prevalence (%)**	**No. examined**	**Prevalence (%)**
2003	1272.686	0.849	18261	1.659	303	4.29	1001	0.3
2004	1649.19	0.374	25750	4.726	1217	5.177	988	0.405
2005	1110.31	0.438	27933	4.493	1255	1.673	834	0.959
2006	758.051	0.616	42537	2.591	1102	2.904	641	0.624
2007	516.948	0.263	43907	1.635	718	3.9	708	0.282
2008	97.186	0.268	16940	1.305	221	4.977	542	0

**Figure 2:**
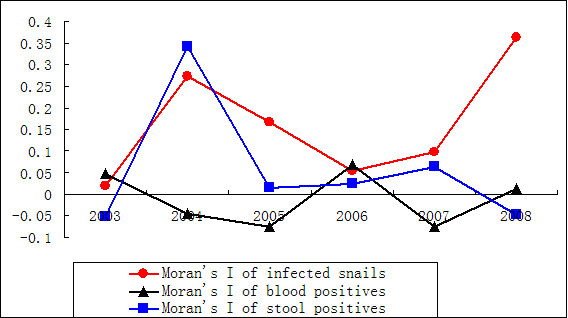
**The dynamic change of Moran’s I value of *S. japonicum*-infected snails, human sero-prevalence and human stool prevalence in the study villages of Jiangsu province, China, from 2003 to 2008.**

The results of the global autocorrelation statistics for infected snails, human sero-prevalence, and human stool prevalence in each year are summarized in Table 2. The results of the global Moran’s I tests were statistically significant (z-score greater than 1.96) and indicate spatial heterogeneity. The global autocorrelation statistics for infected snails in 2004 and 2008 and stool prevalence in 2004 were also statistically significant. The spatial autocorrelation is graphically depicted in Figure 2. Results show that the change in spatial autocorrelation of the sero-prevalence was relatively stable with intervals between −0.075 and 0.069. Between 2004 and 2007, the spatial correlation of the infected snail populations fell dramatically from 0.272 to 0.098, and that of the stool prevalence also decreased from 0.342 to 0.063. After 2007, a rapid increase was observed for the infected snails and a further decrease for the stool prevalence.

**Table 2:** **The yearly Moran’s I value of *S. japonicum*-infected snails, sero-prevalence and stool prevalence in the study villages of Jiangsu province, China, from 2003 to 2008**

**Year**	**Infected snails**	**Human sero-prevalence**	**Human stool prevalence**
2003	0.021	0.048	−0.052
2004	0.273 *	−0.045	0.342 *
2005	0.168	−0.074	0.015
2006	0.055	0.069	0.025
2007	0.098	−0.075	0.064
2008	0.363 *	0.014	−0.048

* P<0.05.

**Figure 3:**
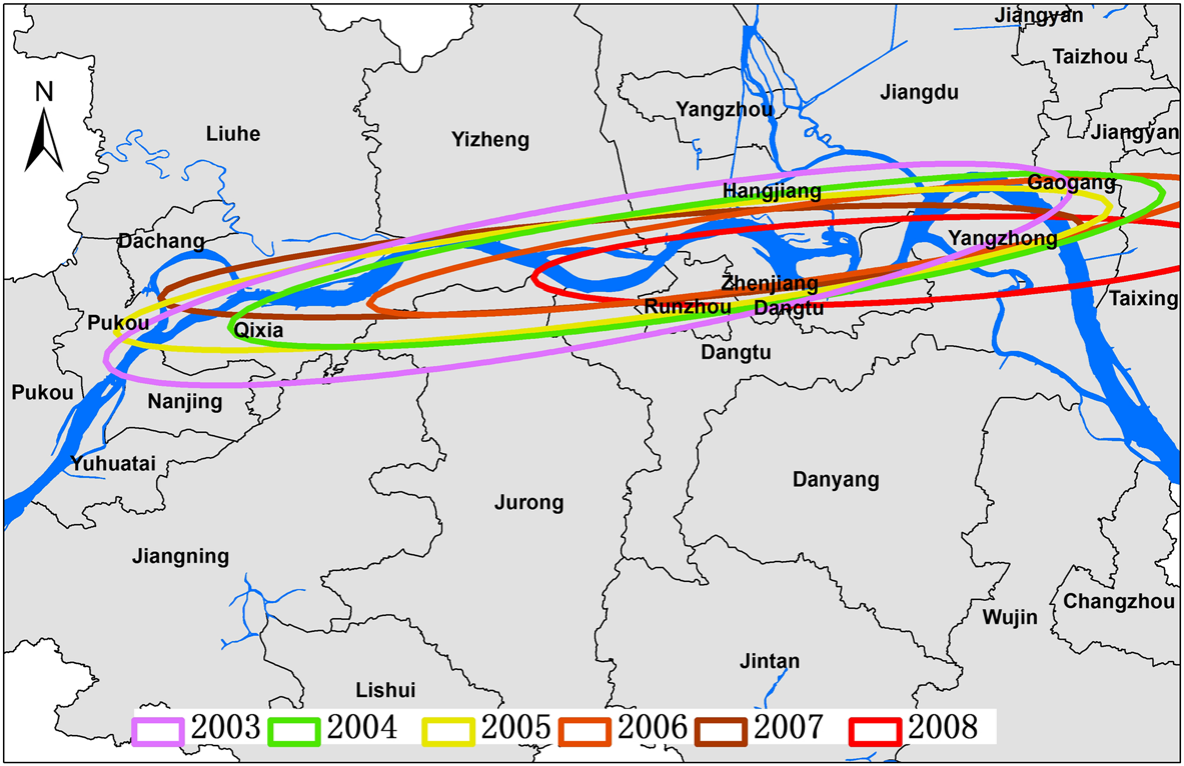
**Directional distribution of *S. japonicum*-infected snails in Jiangsu province, China, from 2003 to 2008.**

Figure 3 shows the series of directional distributions of the infected snails in each year. Their shapes are similar from one year to another, and the ellipses are generally oriented along the Yangtze river. From 2003 to 2008, both the long and short axes became shorter, which means that the standard deviations of the ellipses were decreasing. The central points of the ellipse polygons moved from West to East from 2003 to 2008.

**Figure 4:**
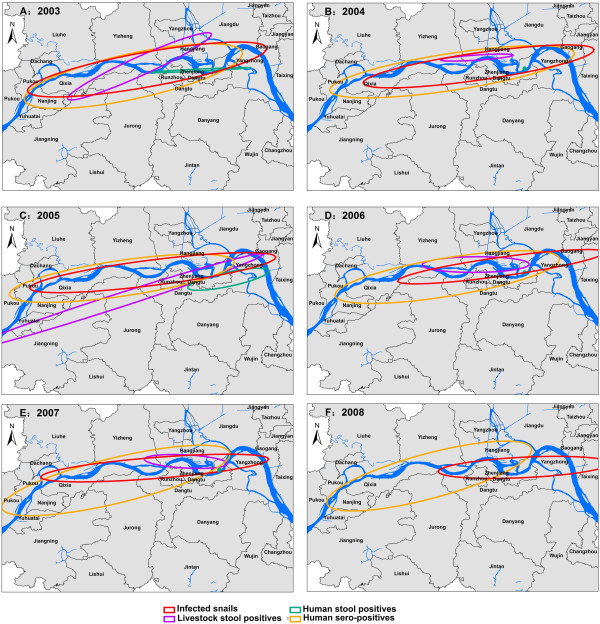
**Directional distribution of *S. japonicum*-infected snails and study factors in the study villages of Jiangsu province, China, in each year from 2003 to 2008.**

The serial comparisons of the directional distribution from 2003 to 2008 are shown in Figure 4A-F. The ellipse polygons of the sero-prevalence retained a relatively stable shape while other polygons changed significantly. The shapes of these ellipse polygons were heterogeneous, with ellipse polygons intersecting in some regions but not in others. These patterns are not suggestive of a significant spatial relationship between infected snails and the investigated variables describing human infection status.

Table 3 presents the spatial error model estimates for the density of infected snails. The spatial regression results show that there was no significant correlation between the density of infected snails and other study factors (*P*>0.05).

**Table 3:** **Spatial error model estimations for the density of *S. japonicum*-infected snails in Jiangsu province, China, from 2003 to 2008**

**Variable**	**2003**	**2004**	**2005**	**2006**	**2007**	**2008**
Constant	0.032*	0.025*	0.019*	0.045*	0.024*	0.015*
Human sero-prevalence	−0.051	0.177	0.001	−0.444	−0.178	−0.195
Human stool prevalence	−0.012	−0.143	−0.084	−0.123	−0.062	−0.013
Livestock prevalence	−0.062	−0.609	0.034	−0.015	−0.215	-**
LAMBDA	0.026	0.423*	0.368*	0.008	0.167	0.002

* P<0.05.

** No infected livestock was found in 2008.

## Discussion

The control of schistosomiasis japonica, similar to the control of any infectious disease, aims to interrupt the parasite lifecycle through interventions intended to eliminate the intermediate host, eliminate the parasite from the definitive host, prevent infection of the intermediate or definitive host, etc. [29,30]. In highly endemic regions, the provision of praziquantel to the local residents is effective at reducing the infection rate [31-33]. However, this does not always interrupt transmission, as throughout history, livestock such as cattle were the main source of infection for schistosomiasis in many areas in China [34,35]. The transmission patterns and spatial distribution of the total and infected snails and the influences of environmental and socio-economic determinants have been considered in a series of epidemiological studies supported by spatial modeling [19].

In Jiangsu province, the area and density of infected snails decreased significantly until 2008. The number of livestock in endemic villages also decreased significantly, to 542 in 2008 from 1001 in 2003. The prevalence in livestock was very low, with no infected livestock found in 2008. However, the prevalence in humans was stable, with no significant difference between serological and stool positive rates over the study period. This might indicate that the contribution from livestock to human infection is not as large anymore as it had been historically.

Moran’s I (Spatial Statistics) measures spatial autocorrelation based on both locations and attribute information, and evaluates whether the pattern expressed is clustered, dispersed, or random [36,37]. In general, a Moran’s Index value near +1.0 indicates clustering while an index value near −1.0 indicates dispersion. Figure 2 shows that the Moran’s I of the sero-prevalence was between −0.075 and 0.069, indicating that the distribution was random and the sero-prevalence stable. The Moran’s I of infected snails increased from 2004 to 2007, indicating that the distribution of infected snails become more and more clustered in some regions. Indeed, the density increased from 0.373 to 0.616 infected snails per 0.1 m^2^, while the area with infected snails was decreasing dramatically. This suggests that schistosomiasis transmission is ongoing in certain areas and that control measures may be forcing transmission into ever smaller refugia (areas ecologically or otherwise most suitable for transmission). The variation curve of the stool prevalence also increased from 2004 to 2007. To determine whether these cluster regions were stable, the directional distribution analysis was carried out.

From 2003 to 2008, the standard deviations of ellipses around infected snail areas were decreasing and the central points of the ellipses moved from West to East, indicating that the habitats of infected snails had become smaller, and that clusters existed in special regions. The serial ellipse polygons in Figure 3A-F indicate that these spatial distributions were significantly different from each other, but the spatial correlation between infected snails and other study factors was not significant. The ellipse polygons overlap in some regions in each year, and studied factors appear to contribute to the distribution of infected snails in some regions. After integrating the spatial error model, we found that the relationship between the study factors and the spatial distribution of infected snails was not strong, confirming data from field studies. For example, no infected livestock or wild mice were detected in the infected snail habitats [38].

Limitations of the current study must also be recognized. First, the considered explanatory factors for infected snails were limited to the serological and parasitological status of the local human and livestock population, while the spatial distribution of infected snails may also depend on other factors, which consequently should be taken into account to improve model accuracy. Second, we explored the risk factors using retrospective data. Third, the sensitivity and specificity of serological and stool tests are not perfect [39,40].

## Conclusions

In conclusion, the contribution of the local infection sources including humans and livestock to the distribution of infected snails may not be significant, and external factors need further study, e.g. temporal migration from other endemic areas. It also appears that snail control may be restricting infected snails into smaller yet ecologically more suitable areas for transmission. The present study describes a way to identify risk factors through retrospective study and spatio-temporal analysis, and such a framework could assist in designing evidence-based control strategies in the process of schistosomiasis elimination.

## Competing interests

The authors have declared that no competing interests exist.

## Authors’ contributions

Conceived and designed the experiments: KY YSL. Performed the experiments: KY WL LPS YXH JFZ FW DRH. Analyzed the data: KY WL. Contributed reagents/materials/analysis tools: KY WL. Wrote the paper: KY PS. All authors read and approved the final version of the manuscript.
